# Blind Channel and Data Estimation Using Fuzzy Logic-Empowered Opposite Learning-Based Mutant Particle Swarm Optimization

**DOI:** 10.1155/2018/6759526

**Published:** 2018-12-06

**Authors:** Muhammad AsadUllah, Muhammad Adnan Khan, Sagheer Abbas, Atifa Athar, Syed Saqib Raza, Gulzar Ahmad

**Affiliations:** ^1^Department of Computer Science, National College of Business Administration and Economics, Lahore, Pakistan; ^2^Department of Computer Science and IT, The University of Lahore, Lahore, Pakistan; ^3^Department of Computer Science, CUI, Lahore, Pakistan

## Abstract

Multiple-input and multiple-output (MIMO) technology is one of the latest technologies to enhance the capacity of the channel as well as the service quality of the communication system. By using the MIMO technology at the physical layer, the estimation of the data and the channel is performed based on the principle of maximum likelihood. For this purpose, the continuous and discrete fuzzy logic-empowered opposite learning-based mutant particle swarm optimization (FL-OLMPSO) algorithm is used over the Rayleigh fading channel in three levels. The data and the channel populations are prepared during the first level of the algorithm, while the channel parameters are estimated in the second level of the algorithm by using the continuous FL-OLMPSO. After determining the channel parameters, the transmitted symbols are evaluated in the 3rd level of the algorithm by using the channel parameters along with the discrete FL-OLMPSO. To enhance the convergence rate of the FL-OLMPSO algorithm, the velocity factor is updated using fuzzy logic. In this article, two variants, FL-total OLMPSO (FL-TOLMPSO) and FL-partial OLMPSO (FL-POLMPSO) of FL-OLMPSO, are proposed. The simulation results of proposed techniques show desirable results regarding MMCE, MMSE, and BER as compared to conventional opposite learning mutant PSO (TOLMPSO and POLMPSO) techniques.

## 1. Introduction

In the field of communication systems, the wireless communication branch is rapidly growing, and fast technology developments are needed to meet the requirement. Wireless communication uses wireless channels instead of wireline channels. The rapid growth of the wireless communication system needs technological advances. Wireless connection provides a variety of services ranging from voice to data and multimedia. Due to the physical properties of the channel, the signal is affected, and unwanted effects occur in wireless communication. Interaction of wireless signals with the environment is very complex. Some problems happen on the channel between the transmitter and receiver because of large objects, diffraction of the electromagnetic waves around obstructing objects, and also signal scattering. Due to these interactions, the signal arriving at the receiver copes with different attenuation, distortion, delays, and phase shift. The inference of these multipaths may be constructive or destructive. The signal power can be slightly diminished when the destructive interface occurs.

It is essential for optimum performance of wireless communication systems to provide accurate channel state information (CSI) for coherent detection of the signal received at the receiver end. A noncoherent method differential demodulation technique is used for demodulation and detection of the transmitted signal when CSI is not available at the receiver. The deployment of the noncoherent method costs about 3-4 dB loss in SNR as compared with the coherent detection method. Due to such massive loss by the noncoherent detection method, research directed toward coherent detection for providing CSI at the receiver in wireless communication systems [1, 2].

Multiuser detection (MUD) as the receiver technology uses compressive sensing (CS) for the detection of inferring signals. If most of the devices are not in the active state, then the transmitting signal vector because of a large number of nonzero elements has a sparse property. Hence, decoding of the transmitted signal would become a compressive signal problem. For a system that has a small number of high activity users, the long-term evolution is more suitable [2, 3].

In modern communication systems, the primary issue is to enhance the channel capacity of the system without affecting the service quality of the system. The multiple-input and multiple-output (MIMO) method is found to be effective in enhancing the data rates and resolving the issue of the channel capacity [1, 4–6]. In this method, algorithms are used to estimate the signals at both the sender and the receiver ends of the antennas [7], due to which the data rates increase as well as the bandwidth of the channel capacity [8–11]. Few transmitter antennas and beneficiary radio wires are utilized in this technique to enhance the correspondence technique of the system. The transmit information is calculated on various transmission paths depending on the amount of data conveyed by the MIMO framework increments [12].

On the receiving end, some antennas collect the information received, and different calculations are performed to reassemble the information and reestablish the data at the receiver's end accordingly. Due to the increment in the range and the amount of the information without any additional transmitting power or the data transfer capacity, the MIMO innovation is considered as the midpoint for remote communication [13, 14].

The medium MIMO innovation technique can also be utilized along with multicarrier code-division multiple access (MC-CDMA) and orthogonal frequency-division multiplexing (OFDM) to enhance the significant volume growth for numerous correspondences [7–9].

The maximum likelihood (ML) method is one of the optimal detectors in MUD, but ML is complicated to use it for achievement of exponential complexity. In a less-complicated situation, the suboptimal MUD detectors like the zero-forcing or null-steering detector, minimum mean square error (MMSE) detector in M2M, and maximum a posteriori or marginal likelihood detectors are used. The primary concern of the multiuser detection is based on the knowledge of strategies to demodulate the data sent simultaneously by several servers to share a multiaccess channel. The last two suboptimal approaches use matrix inversion and also are very simple. Some evolutionary algorithms like repeated weighted boosting search (RBS), fuzzy adaptive differential evolution (FADE), and differential evolution algorithms (DEAs) are helpful for channel estimation (CE) and multiuser detection [6]. For the CE problem, the continuous search space is used, and for multiuser detection, the discrete search space is used, and for improvement of the spectral efficiency multiuser-MIMO (MU-MIMO), broadcasting approaches are mostly used [10, 13]. At the transmitter because of course knowledge of channel state information, the quality of transmitting precoding to dominate the multiuser inference degraded [10]. Therefore, the system throughput may get affected by the interface from coscheduled user equipment.

The alternate emerging numerous strategies like particle swarm optimization (PSO) [15], partial opposite mutant particle swarm optimization (POMPSO), total opposite mutant particle swarm optimization (TOMPSO) [7, 9, 10], genetic algorithm (GA), island GA, differential equation (DE), and island DE can be used to further enhance the performance of the digital communication system [15]. In this article, we performed the channel estimation for high data rates in correspondence to both the sender and the receiver ends. As some distortion adds up to the signal during communication through the channel, the signal strength weakens and the receiver end might not be able to collect the accurate information. To overcome this issue, fuzzy logic is implemented to improve the data and channel estimation process [9, 10]. In this article, fuzzy logic empowered the opposite particle swarm optimization-based new variant for the communication system and implemented it using the PSO technique.

In this research work, we consider the MIMO system that consists of different numbers of users. It also assumed that the channel is flat fading and cyclostationary. The main contributions of the paper are listed as follows:

- We formulate an optimization problem in which the objective is to minimize the MMSE and BER.
- A fuzzy logic-empowered opposite learning-based mutant particle swarm optimization (FL-OLMPSO) algorithm has been proposed for the estimation of the user data and the channel coefficients.
- We compare our proposed method with other studied algorithms like TOMPSO and POMPSO in the literature. Simulation results show that the proposed algorithms give attractive results as compared to different algorithms.

The rest of the paper is organized as follows: the MIMO system model is explained in Section 2. The FL-OLMPSO-based optimization problem is formulated in Section 3.  Section 4 presents the simulation results and discussion. Finally, the research work is concluded in Section 5.

## 2. System Model

There are *A* transmitting antennas and *B* receiving antennas. The flat fading channel is implemented. The channel is expected to be stationary during the communication process of *Q* symbols. The received signal at the receiver antenna *b* is as follows [1]:

(1) $$\begin{array}{c} r_{\text{b}}\left(i\right)=\overset{A}{\underset{a=1}{\sum }}h_{b.a}d_{a}\left(i\right)+v_{b}\left(i\right), \end{array}$$

where *i* is the index of the symbol, *h*_*b*.*a*_ is the flat fading channel coefficient that links the transfer antenna *a* to the receiver antenna *b*, *d*_*a*_(*i*) is the *i*th symbol transmitted from the antenna *a* taking value from the symbol set {−1,+1} of binary phase shift key (BPSK), and *v*_*b*_(*i*) is the additive white Gaussian noise (AWGN) with *E*[|*v*_*b*_(*i*)|^2^]=2*σ*_*V*_^2^.

The following MIMO channel equation will represent the complete system:

(2) $$\begin{array}{c} r\left(i\right)=\mathrm{Hd}\left(i\right)+v\left(i\right), \end{array}$$

where **v**(**i**) represents AWGN:

(3) $$\begin{array}{c} v\left(i\right)={\left[v_{1}\left(i\right)\;\;v_{2}\left(i\right)\ldots v_{B}\left(i\right)\right]}^{\text{T}}. \end{array}$$

The transmitted symbol vector is

(4) $$\begin{array}{c} d\left(i\right)={\left[d_{1}\left(i\right)\;\;d_{2}\left(i\right)\ldots d_{A}\left(i\right)\right]}^{\text{T}}, \end{array}$$

and the received signal vector is

(5) $$\begin{array}{c} r\left(i\right)={\left[r_{1}\left(i\right)\;\;r_{2}\left(i\right)\ldots r_{B}\left(i\right)\right]}^{\text{T}}. \end{array}$$

The channel gain at the receiver antenna can always be normalized to unity:

(6) $$\begin{array}{c} \overset{A}{\underset{a=1}{\sum }}{\left|h_{b,a}\right|}^{2}=1, \end{array}$$

where *H*(*b*, *a*)=*h*_*b*.*a*_.

Now define a received data matrix with *B* × *V* dimensions and transmitted data matrix with *A* ∗ *V* dimensions as follows [1]:

(7) $$\begin{array}{c} R=\left[r\left(1\right)\;\;r\left(2\right)\ldots \;r\left(Q\right)\right], \end{array}$$

(8) $$\begin{array}{c} D=\left[d\left(1\right)\;d\left(2\right)\ldots \;d\left(Q\right)\right], \end{array}$$

respectively. Then, the PDF of the received signal matrix **R** conditioned on the MIMO channel matrix **H** and the transmitted data matrix **D** can be written as follows:

(9) $$\begin{array}{c} P_{\text{rob}}\left(\frac{R}{H,D}\right)=\frac{1}{2\pi \sigma_{v}^{2}BQ}e^{-\left(1/2\sigma_{v}^{2}\right)}\overset{Q}{\underset{i=1}{\sum }}{\left|\left|r\left(n\right)-Hd\left(i\right)\right|\right|}^{2}. \end{array}$$

The ML estimation of the transmitted symbols **D** and the MIMO channel matrix **H** can be obtained by maximizing *P*_rob_(**R**/(**H**, **D**)) over **H** and **D** mutually. Equally, the joint ML estimation can be obtained by minimizing the following cost function:

(10) $$\begin{array}{c} J_{\text{ML}}\left(\tilde{D},\tilde{H}\right)=\frac{1}{B\;\;x\;\;Q}\overset{Q}{\underset{i=1}{\sum }}\left|\left|r\left(i\right)-\tilde{H}\tilde{d}\left(i\right)\right|\right|{}^{2}. \end{array}$$

Namely, the joint ML CDE is obtained as follows:

(11) $$\begin{array}{c} J_{\text{ML}}\left(\tilde{D},\tilde{H}\right)=\arg\left\{\underset{\tilde{S},\tilde{H}}{\min}J_{\text{ML}}\left(\tilde{D},\tilde{H}\right)\right\}. \end{array}$$

Equation (10) demonstrates that the search for the optimal joint ML solution is over the discrete space of the transmitted symbols and the continuous space of the MIMO channel matrix mutually.

### 2.1. Improved Cost Function

Equation (10) can be written as follows:

(12) $$\begin{array}{c} J_{\text{ML}}\left(\tilde{D},\tilde{H}\right)=\frac{1}{B\;*\;Q}\left[\overset{Q}{\underset{i=1}{\sum }}r^{2}\left(i\right)-2\overset{Q}{\underset{i=1}{\sum }}r\left(i\right)\tilde{H}\tilde{d}\left(i\right)+\overset{Q}{\underset{i=1}{\sum }}{\left|\left|\tilde{H}\tilde{d}\left(i\right)\right|\right|}^{2}\right]\\ =\frac{1}{B\;*\;Q}\left[\overset{Q}{\underset{i=1}{\sum }}r^{2}\left(i\right)-\left\{2\overset{Q}{\underset{i=1}{\sum }}r\left(i\right)\tilde{H}\tilde{d}\left(i\right)+\overset{Q}{\underset{i=1}{\sum }}{\left|\left|\tilde{H}\tilde{d}\left(i\right)\right|\right|}^{2}\right\}\right], \end{array}$$

where *B* represents the receiver antennas and *Q* symbols are transmitted. It is also shown that **H** and **D** accrue in second and third terms. Then, we let

(13) $$\begin{array}{c} C_{\text{ML}}\left(\tilde{D},\tilde{H}\right)=2\overset{Q}{\underset{i=1}{\sum }}r\left(i\right)\tilde{H}\tilde{d}\left(n\right)-\overset{Q}{\underset{i=1}{\sum }}{\left|\left|\tilde{H}\overset{ˇ}{d}\left(i\right)\right|\right|}^{2}. \end{array}$$

Substituting the values from equation (13) in equation (12), we get

(14) $$\begin{array}{c} J_{\text{ML}}\left(\tilde{D},\tilde{H}\right)=\frac{1}{B\;\;x\;\;Q}\left[\overset{Q}{\underset{i=1}{\sum }}r^{2}\left(i\right)-C_{\text{ML}}\left(\tilde{D},\tilde{H}\right)\right]. \end{array}$$

Equation (12) can be written as follows:

(15) $$\begin{array}{c} J_{\text{ML}}\left(\tilde{D},\tilde{H}\right)=\left[\underset{\tilde{D},\overset{ˇ}{H}}{\min}\left[\overset{Q}{\underset{i=1}{\sum }}r^{2}\left(i\right)-C_{\text{ML}}\left(\tilde{D},\tilde{H}\right)\right]\right]. \end{array}$$

It means the joint ML CDE can be written as follows:

(16) $$\begin{array}{c} J_{\text{ML}}\left(\tilde{D},\tilde{H}\right)=\underset{\tilde{\tilde{D}},\overset{ˇ}{\tilde{H}}}{\max}C_{\text{ML}}\left(\tilde{D},\tilde{H}\right). \end{array}$$

In this article, we have consigned fuzzy logic-empowered opposite learning mutant particle swarm optimization (FL-OLMPSO) for the joint channel and symbol estimation for the MIMO system. We have used three-layered methods. At one layer, a continuous version of FL-OLMPSO was exploited, and at the next layer, a soft version of discrete FL-OLMPSO was applied as shown in Table 1. FL-OLMPSO is the updated version of the OLMPSO algorithms proposed by Khan et al. [10].

The accumulative function (15) is considered as fitness function of the MIMO system and is used to compute the performance of the proposed algorithm as shown in Table 1.

## 3. Proposed Fuzzy Logic-Empowered Opposite Learning Mutant Particle Swarm Optimization (FL-OLMPSO)

Fuzzy logic-based opposite mutant PSO is used in which velocity of the particle is updated using the fuzzy logic controller taking two inputs: local intelligence and global intelligence, on the bases of these input parameters and giving the updated velocity of the particle as shown in Tables 1–3.

Mathematically and graphically I/O variables membership functions (MFs) which are used in updating the velocity of the swarm given in the proposed FL-OLMPSO are shown in Table 2.

The fuzzy system consists of four core components. They are fuzzy prepositions, lookup table, inference engine, and defuzzifier as shown in the following sections.

Sections 3.1to 3.4 describe how we update the velocity using the fuzzy logic system in detail.

### 3.1. Fuzzy Prepositions

A fuzzy compound proposition is an alignment of minute fuzzy propositions using the connectives “or,” “and,” and “not” which represent the fuzzy union, intersections, and complement, respectively. Here, *l*, *g*, *p*_v_, and *U*_v_ variables represent local intelligence, global intelligence, previous velocity, and updated velocity. Then, the following fuzzy propositions hold:

(17) $$\begin{array}{c} t:l\times g\times p_{\text{v}}⟶U_{\text{v}}. \end{array}$$

All input and output variable values are mapped from real ranges to probability ranges because the fuzzy expert system works on probability (range 0-1).

Here, the function *t*-norm for the final layer in equation (17) is defined as follows:

(18) $$\begin{array}{c} t:\left[0,1\right]\times \left[0,1\right]\times \left[0,1\right]⟶\left[0,1\right]. \end{array}$$

Equation (18) transforms the membership functions of fuzzy sets of local intelligence, global intelligence, previous velocity, and updated velocity for a final layer of the proposed fuzzy inference system among membership functions of the intersection of local intelligence, global intelligence, previous velocity, and updated velocity, that is,

(19) $$\begin{array}{c} t\left[\mu_{\text{L}}\left(l\right),\mu_{\text{G}}\left(g\right),\mu_{P_{\text{v}}}\left(P_{\text{v}}\right)\right]=\min\left[\mu_{\text{L}}\left(l\right),\mu_{\text{G}}\left(g\right),\mu_{P_{\text{v}}}\left(p_{\text{v}}\right)\right]\;\;. \end{array}$$

In equation (19), for the function *t* to get qualified as an intersection, the following axioms must be satisfied and the function will be called as *t*-norm:

*Axiom t1*. Bounded condition:

(20) $$\begin{array}{c} t\left(0,0\right)=0;t\left(\Omega ,1\right)=t\left(1,\Omega \right)=\Omega . \end{array}$$

*Axiom t2*. Commutativity:

(21) $$\begin{array}{c} t\left(\Upsilon ,\;\;\Gamma \right)=t\left(\Gamma ,\;\;\Upsilon \right). \end{array}$$

*Axiom t3*. Nondecreasing:

(22) $$\begin{array}{c} \text{if}\;\;\Upsilon \leq \Upsilon^{\prime }\;\;\mathrm{and}\;\;\Gamma \leq \Gamma^{\prime },\;\;\mathrm{then}\;\;t\left(\Upsilon ,\Gamma \right)\leq t\left(\Upsilon^{\prime },\Gamma^{\prime }\right). \end{array}$$

*Axiom t4*. Associativity:

(23) $$\begin{array}{c} t\left[t\left(\Omega ,\Upsilon \right),\Gamma \right]=t\left[\Omega ,t\left(\Upsilon ,\Gamma \right)\right]. \end{array}$$

Equation (19) can be written regarding *t*-norm as follows:

(24) $$\begin{array}{c} \mu_{\text{L}\cap \text{G}\cap P\text{v}}\left(l,g,p_{\text{v}}\right)=t\left[\mu_{\text{L}}\left(l\right),\mu_{\text{G}}\left(g\right),\mu_{P_{\text{v}}}\left(p_{\text{v}}\right)\right]. \end{array}$$

From equations (19) and (24),

(25) $$\begin{array}{c} \mu_{\text{L}\cap \text{G}\cap P\text{v}}\left(l,g,p_{\text{v}}\right)=\min\left[\mu_{\text{L}}\left(l\right),\mu_{\text{G}}\left(g\right),\mu_{P_{\text{v}}}\left(p_{\text{v}}\right)\right]. \end{array}$$

### 3.2. Lookup Table

The lookup table for the proposed FL-OLMPSO contains 10 input-output rules from 80 as shown in Table 3.

Fuzzy IF-THEN rules are the conditional statement applied to the membership functions. These rules are elements of the fuzzy rule base. Others components like the rules surface and rules viewer are dependent upon the fuzzy rule base, so the fuzzy rule base is a major element of FIS. The fuzzy rule base of our expert system has 80 rules. Rules are denoted by *Rv*^*η*^, where  1 ≤ *η* ≤ 80.

 
*Rv*^1^ = **IF** local intelligence is small AND global intelligence is small AND previous velocity is very slow, **THEN** updated velocity is very slow
 
*Rv*^2^ = **IF** local intelligence is medium AND global intelligence is small AND previous velocity is slow, **THEN** updated velocity is slow
 
*Rv*^3^ = **IF** local intelligence is medium AND global intelligence is medium AND previous velocity is slow, **THEN** updated velocity is medium
 
*Rv*^80^ = **IF** local intelligence is large AND global intelligence is large AND previous velocity is fast, **THEN** updated velocity is very fast

### 3.3. Inference Engine

Fuzzy inference is the way toward mapping from an offered contribution to a yield utilizing fuzzy logic. The main component of fuzzy inference is MFs, FL operators, and IF-THEN rules. A single fuzzy relation is created by all rules in the fuzzy rule base. It lies under the inner product on the input which can be seen as an only fuzzy IF-THEN rule.

All rules in the fuzzy rule base are combined into a single fuzzy relation that lies under the inner product on input universes of discourse, which is then viewed as an only fuzzy IF-THEN rule.

Let *Rv*^*η*^ be a fuzzy relation that represents the fuzzy IF-THEN rule of the final layer of the proposed FL-OLMPSO expert system, which is

(26) $$\begin{array}{c} Rv^{\eta }=L^{\eta }\times G^{\eta }\times P_{\text{v}}^{\eta }⟶U_{\text{v}}^{\eta }. \end{array}$$

Equation (26) can be written as follows:

(27) $$\begin{array}{c} \mu_{\text{L}\cap \text{G}\cap Pv}\left(l,g,p_{\text{v}}\right)=\left[\mu_{\text{L}}\left(l\right)\cap \mu_{\text{G}}\left(g\right)\cap \mu_{P_{\text{v}}}\left(p_{\text{v}}\right)\right]. \end{array}$$

The rules of the final layer are interpreted as a single fuzzy relation defined by

(28) $$\begin{array}{c} R_{80}=\overset{80}{\underset{\eta =1}{\cup }}Rv^{\eta }. \end{array}$$

This combination of rules is called the Mamdani combination. Assume *i* and Ψ be any two fuzzy sets and also the input and output of the fuzzy inference engine, respectively. To view *R*_80_ as a single fuzzy IF-THEN rule by using the comprehensive modus ponens, we obtain the output of the FIE as follows:

(29) $$\begin{array}{c} \mu_{\text{very slow}\cap \text{slow}\cap \text{medium}\cap \text{fast}\cap \text{very fast}}\left(\Psi \right)=\sup_{i\in \left(L,G,P_{v}\right)}t\left[\mu_{i}\left(l,g,p_{\text{v}}\right),\;\;\mu_{R_{80}}\left(l,g,p_{v},U_{\text{V}}\right)\right]. \end{array}$$

The product inference engine (PIE) of the proposed FL-OLMPSO expert system can be written as follows:

(30) $$\begin{array}{c} \mu_{\xi \;\;}\text{updated velocity}=\underset{1\leq \eta \leq 80}{\max}\left[\sup_{i\in \left(L,G,P_{\text{v}}\right)}\left(\overset{80}{\underset{j=1}{\prod }}\left(\mu_{L,G,P_{v}}\left(L,G,P_{v}\right),\mu_{A_{1i}A_{2i},A_{3i}}\left(a_{1},a_{2},a_{3}\right)\right)\right)\right]. \end{array}$$

### 3.4. Defuzzifier

One of the most essential components of an expert system is the defuzzifier. It carries out the process of mapping the fuzzy sent to the crisp output. There are three types of the defuzzifier: center of gravity (CoG) defuzzifier, center of average defuzzifier, and maximum defuzzifier. From these, the best defuzzifier is the “center of gravity defuzzifier.” In the proposed FL-OLMPSO-based system, the CoG defuzzifier is used. The CoG defuzzifier specifies ₤^∗^ as the center of the area covered by the MF of Ψ, that is,

(31) $$\begin{array}{c} {₤}^{*}=\frac{\int \xi \;\;\mu_{\xi \;\;}\left(\xi \;\;\right)d\xi \;\;}{\int \mu_{\xi \;\;}\left(\xi \;\;\right)d\xi \;\;}. \end{array}$$

**Figure 1:**
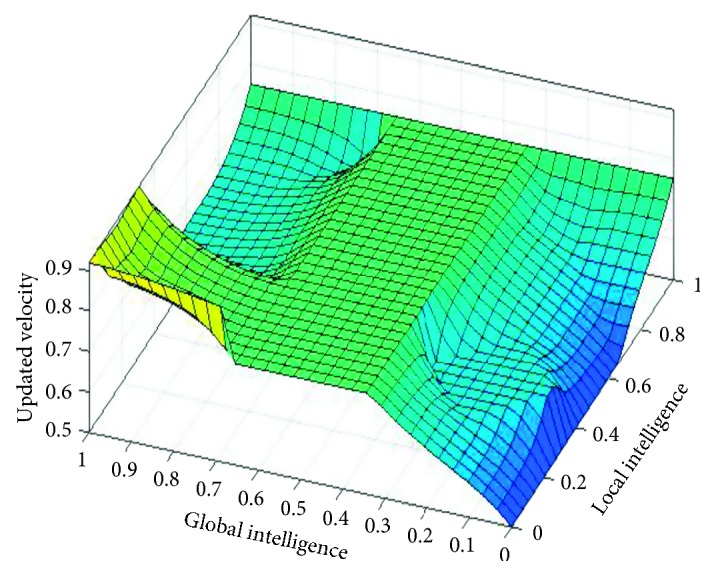
Rule surface for updated velocity of local intelligence and global intelligence.

**Figure 2:**
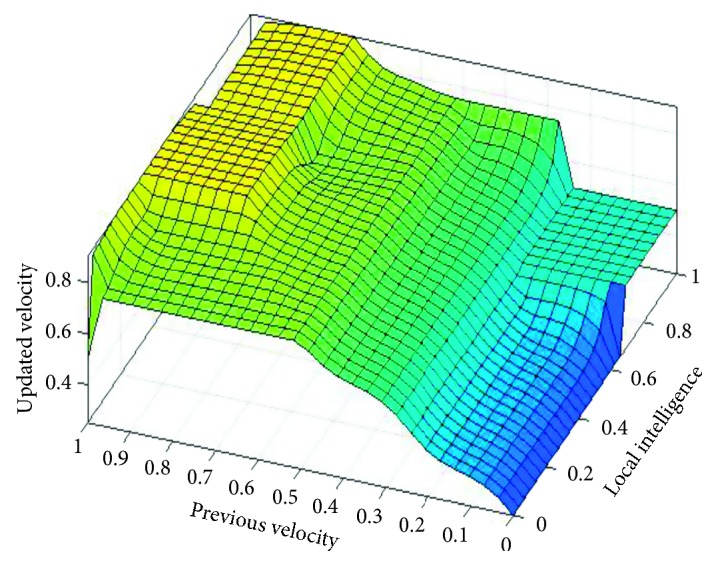
Rule surface for updated velocity based on local intelligence and previous velocity.

**Figure 3:**
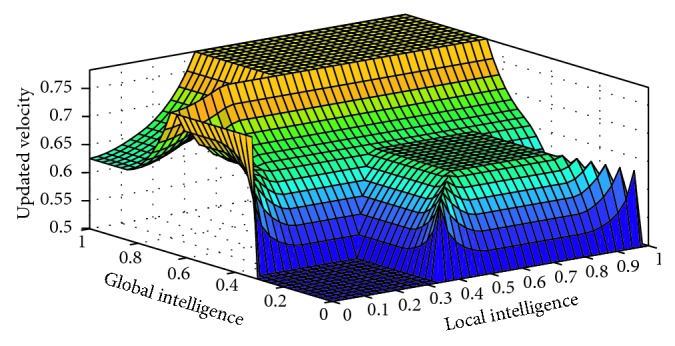
Rule surface for updated velocity based on global intelligence and previous velocity.

The graphical representation of the defuzzifier of the proposed FL-OLMPSO-based system is shown in Figures 1–3.  Figure 1 shows that if local intelligence is small to medium and global intelligence is small, then updated velocity is very slow. If local intelligence is small to medium and global intelligence is small, then updated velocity is slow. If local intelligence is between medium and large and global intelligence is medium, then updated velocity is slow to medium. If local intelligence is large and global intelligence is medium to large, then updated velocity is fast. If local intelligence is very large and global intelligence is very large, then updated velocity is very fast.

Figure 2 shows that if local intelligence is slow to medium and previous velocity is very slow to slow, then updated velocity is very slow. If local intelligence is large to very large and previous velocity is slow to medium, then updated velocity is slow. If local intelligence is medium to large and previous velocity is medium, then updated velocity is medium. If local intelligence is medium and previous velocity is medium to fast, then updated velocity is fast. If local intelligence is large to very large and previous velocity is breakneck, then updated velocity is very fast.

If global intelligence is medium to large and previous velocity is very slow, then updated velocity is very slow. If global intelligence is medium and previous velocity is slow, then updated velocity is slow. If global intelligence is between medium and above and previous velocity is medium to fast, then updated velocity is medium. If global intelligence is large and updated velocity is fast to very fast, then updated velocity is fast. If global intelligence is large to very large and previous velocity is very fast, then updated velocity is very fast as shown in Figure 3.

### 3.5. Lookup Diagrams

**Figure 4:**
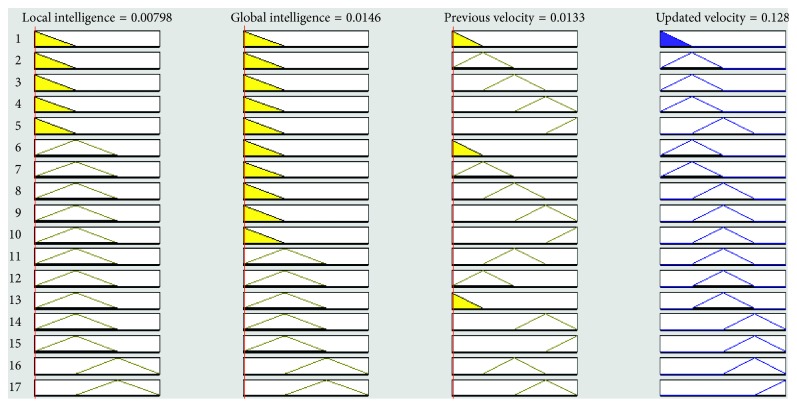
Lookup diagram showing that updated velocity is very slow for the proposed FL-OLMPSO.

**Figure 5:**
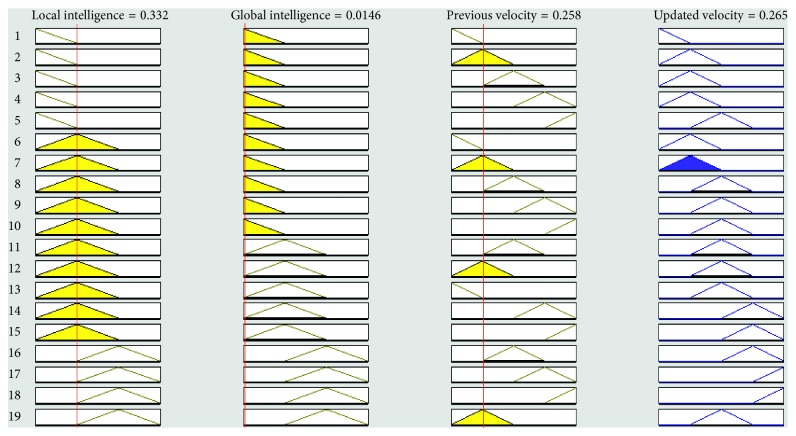
Lookup diagram showing that updated velocity is slow for the proposed FL-OLMPSO.

**Figure 6:**
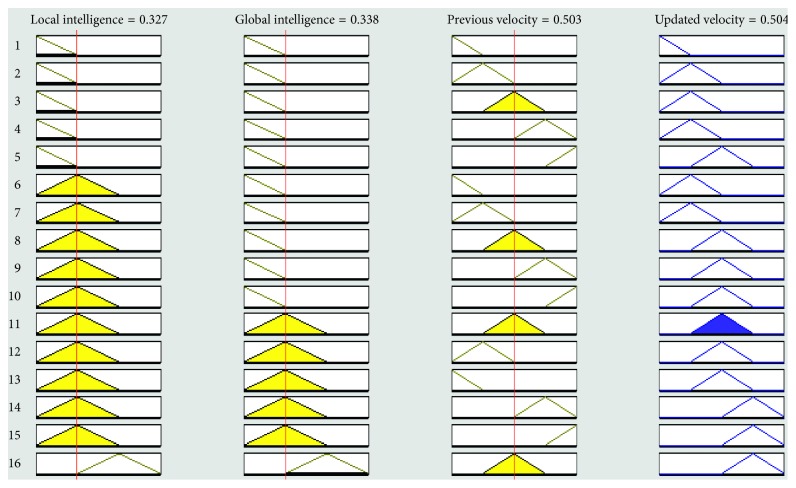
Lookup diagram showing that updated velocity is medium for the proposed FL-OLMPSO.

**Figure 7:**
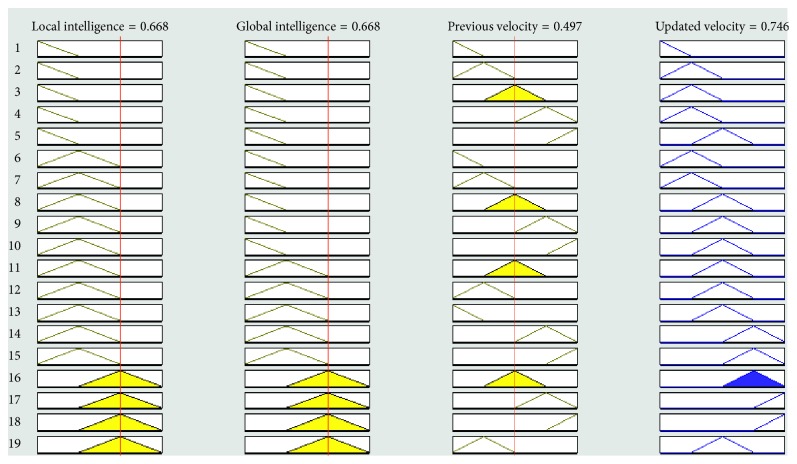
Lookup diagram showing that updated velocity is fast for the proposed FL-OLMPSO.

**Figure 8:**
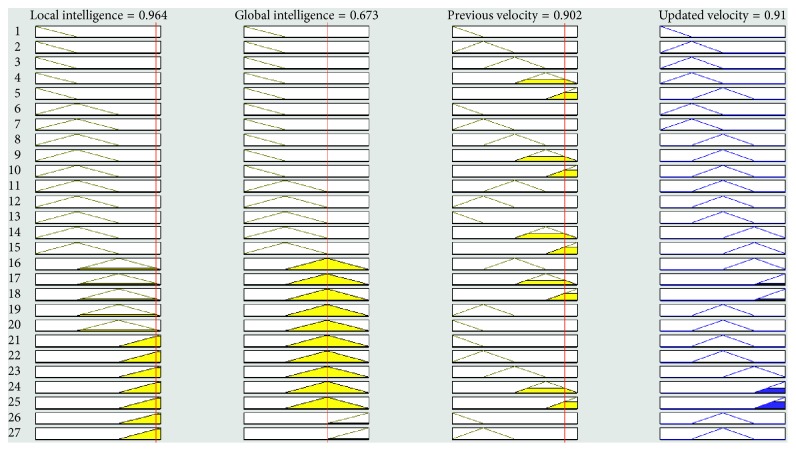
Lookup diagram showing that updated velocity is very fast for the proposed FL-OLMPSO.

Figures 4–8 show the lookup diagrams of the proposed fuzzy logic-empowered opposite learning-based mutant swarm optimization with all possible cases of the updated velocity.

Figure 4 shows that if the local intelligence value is small, the global intelligence value is small, and previous velocity is very slow, then the updated velocity is very slow.

Figure 5 shows that if the local intelligence value is medium, the global intelligence value is small, and previous velocity is slow, then the updated velocity is also slow.

Figure 6 shows that if both (local and global) intelligence values are medium and previous velocity is medium, then the updated velocity is also medium.

Figure 7 shows that if both (local and global) intelligence values are high and previous velocity is medium, then the updated velocity is fast.

Figure 8 shows that if local intelligence is high, global intelligence is medium, and previous velocity is very fast, then the updated velocity is also very fast.

## 4. Results and Discussion

The CDE-MIMO system is implemented using binary phase shift key signalling, and the data sequences length was *Q* = 50. The transmitters are each outfitted with *A* = 3 transmit antennas, while the base station has *B* = 3 antennas. The Rayleigh selective fading channel was implemented in four parts. The Doppler frequency of 25 Hz corresponds to the transmitter using 900 MHz carrier frequency, moving at a speed of 30 km/h. The channel taken for simulation is a 3 ∗ *k* MIMO with *k* = 10 users, data populace is 100, and NoC for both algorithms are 5, as we have chosen the populace size to be 5 ∗ Ph, where Ph is the channel matrix size.

However, in the imitation, the performance can also be measured by minimum mean channel error (MMCE), which is

(32) $$\begin{array}{c} \text{MMCE}=\frac{1}{A*B}\overset{A}{\underset{a=1}{\sum }}\overset{B}{\underset{b=1}{\sum }}\left[h_{\mathrm{a,b}}-{\tilde{H}}^{*}\left(a,b\right)\right]. \end{array}$$

**Figure 9:**
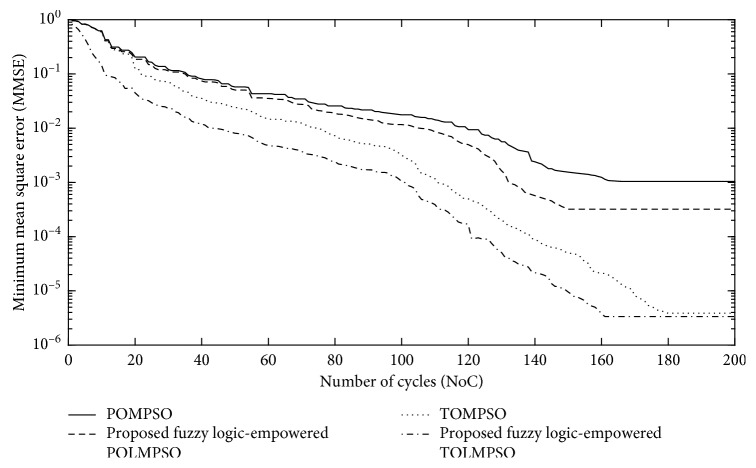
NoC vs MMSE of the proposed FL-OLMPSO with SNR = 25 dB and number of users = 15.

**Figure 10:**
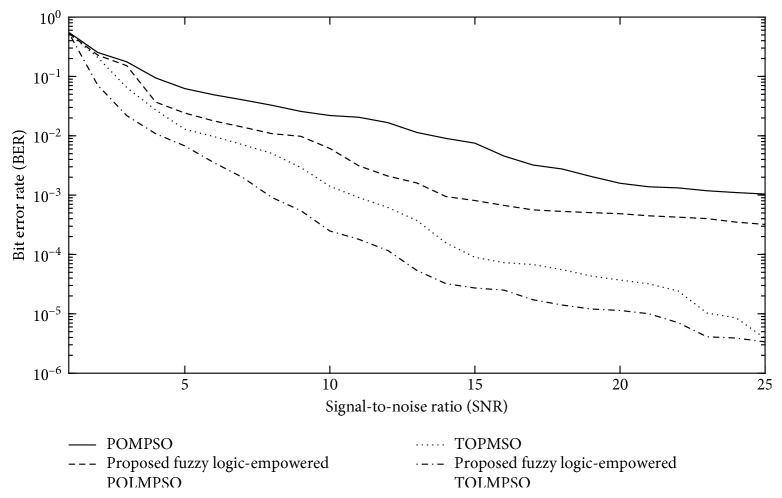
SNR vs BER of the proposed FL-OLMPSO with number of users = 15 and number of cycles (NoC) = 180.

**Figure 11:**
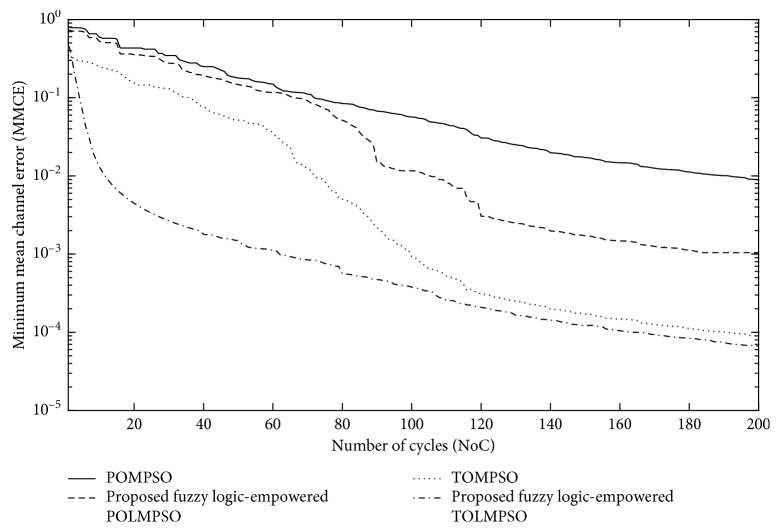
NoC vs MMCE of the proposed FL-OLMPSO with SNR = 25 dB and number of users = 15.

Figures 9 and 10 represent the performance of the proposed FL-OLMPSO for channel and data estimation of the MIMO system in terms of minimum mean square error (MMSE) and bit error rate (BER), while Figure 11 represents the performance of the proposed FL-OLMPSO regarding MMCE, respectively.

Figure 9 shows the number of cycles (NoC) vs MMSE of the proposed FL-OLMPSO-based MIMO system with SNR = 25 dB and the number of users of 15. The 1st and 3rd curves from top to bottom show that POMPSO and TOMPSO converge at 160 and 180 iterations to achieve the MMSE of 10^−3^ and 10^−5.4^, respectively. The 2nd and 4th curves from top to bottom are for the proposed FL-OLMPSO schemes. The 2nd curve from top to bottom shows that the proposed fuzzy logic-empowered partial opposite learning mutant particle swarm optimization (FL-POLMPSO) achieves the MMSE of 10^−3.5^ at 150 iterations. The 4th curve from top to bottom shows that the proposed fuzzy logic-empowered total opposite learning mutant particle swarm optimization (FL-TOLMPSO) achieves the MMSE of 10^−5.5^ at 160 iterations. It can be easily seen that proposed fuzzy logic-based schemes give excellent results as compared to conventional approaches regarding fast convergences as well as MMSE.

Figure 10 shows the signal-to-noise ratio (SNR) vs BER of the proposed FL-OLMPSO-based MIMO system with NoC = 25 dB and the number of users of 15. The 1st and 3rd curves from top to bottom show the conventional POMPSO and TOMPSO schemes, while the 2nd and 4th curves from top to bottom are for the proposed FL-OLMPSO schemes. It can be easily seen that BER comes down by increasing the SNR and proposed fuzzy logic-empowered OLMPSO; both variants give attractive results as compared to conventional OLMPSO variants.

Figure 11 shows the NoC vs MMCE of the proposed FL-OLMPSO-based MIMO system with SNR = 25 dB and the number of users of 15. It can be seen from the 1st and 3rd topmost curves that the conventional schemes (POMPSO and TOMPSO) need 200 iterations to achieve approximately 10^−2^ and 10^−4^, respectively. And 2nd and 4th topmost curves show that proposed fuzzy logic-empowered schemes (FL-POLMPSO and FL-TOLMPSO) need 200 iterations to achieve approximately 10^−3^ and 10^−4.2^, respectively. It means that proposed schemes give better results as compared to conventional systems.

## 5. Conclusion

A blind FL-OLMPSO model has been designed for joint channel and data estimation (CDE). The proposed model is a three-layered model. At the top layer, data and channel population is prepared. At the next level, parameters of the channel are estimated, and at the last level, these parameters are used along with discrete FL-OLMPSO for estimation of transmitted symbols. This article presents two variants of fuzzy logic-based opposite learning mutant particle swarm optimization methods. The performance of the proposed fuzzy logic-based opposite learning mutant PSO (FL-OLMPSO) is evaluated in comparison with that of other swarm algorithms in the literature. Moreover, it is seen that, due to the included fuzzy logic-based velocity factor and opposite-based learning of the swarm, the FL-TOLMPSO gives attractive results regarding MMSE, BER, and MMCE.

## Figures and Tables

**Table 1:** Proposed fuzzy logic-empowered opposite learning mutant particle swarm optimization (FL-OLMPSO) algorithm.

S. no.	Steps	
*Level 1*		
1	Start	
2	2.1. Initialization of data populaces **D**_**p**_ = { **D**_p1_, **D**_p2_, .................. **D**_pa_} and velocity **W**_**d**_	
	2.2. Initialization of channel populaces **D**_**k**_ = { **D**_k1_, **D**_k2_, .................. **D**_ka_} and velocity **W**_**k**_	
3	Compute the wellness of population utilizing the cost work given in (14)	
4	Compute lower bound value (MB_p_, MB_i_) and upper bound value (HB_p_, HB_i_) from **D**_**p**_ and **D**_**k**_ separately	
	*Calculate the opposite populace*	
5	*For FL-TOLMPSO*	*For FL-POLMPSO*
	5.1. Opposite data population	5.1. Opposite data population
	**OD**_**p**_ = {**OD**_p1_, **OD**_p2_, .................. **OD**_pa_}	**OD**_**p**_ = {**OD**_p1_, **OD**_p2_, .................. **OD**_pa/2_}
	**OD**_pi_ = {OD_pi,1_, OD_pi,2_, .................. OD_pi,M_}	**OD**_pi_ = {OD_pi,1_, OD_pi,2_, .................. OD_pi,M_}
	OD_pi,j_ = MB_p_ + HB_a_ − D_pi,j_	OD_pi,j_ = MB_p_ + HB_a_ − D_pi,j_
	5.2. Opposite channel population	5.2. Opposite channel population
	**OD**_**k**_ = {**OD**_k1,_**OD**_k2_, .................. **OD**_ka_}	**OD**_**k**_ = {**OD**_k1,_**OD**_k2_, .................. **OD**_ka/2_}
	**OD**_ki_ = {OD_ki,1_, OD_ki,2_, .................. OD_ki,M_}	**OD**_ki_ = {OD_ki,1_, OD_ki,2_, .................. OD_ki,M_}
	OD_ki,j_ = MB_i_ + HB_i_ − D_ki,j_	OD_ki,j_ = MB + HB_i_ − D_ki,j_
6	Compute the fitness of both opposite populations (**OD**_**p**_ and **OD**_**k**_) using the cost function given in equation (16)	
7	Select the local best particle of the following: 7.1. Data population **M**_bdp_ from **D**_**p**_ and **OD**_**p**_ 7.2. Channel population **L**_bdk_ from **D**_**k**_ and **OD**_**k**_	
8	Select the global best particle of the following: 8.1. Data population **N**_bdp_ = min(**M**_bdp_) 8.2. Channel population **N**_bdp_ = min(**L**_bdp_)	

*Level 2: global best data vector is fixed and continuous FL-OLMPSO algorithm works on the channel population*		
9	Update velocities of each particle of channel population using FIS: W_him(n)_ = W_him(n−1)_ + FLC (LI, GI, W_him(n−1)_)	
10	Update the position of each particle channel population Calculate the mutant operator (MO) M_oh_(i) = ∑_*j*=1_^*k*^(*w*_*hij*_/*k*) D_kim_(n) = D_kim_(n−1) + M_oh_(i) ∗ rand()	
11	Compute the fitness of mutated particles of channel population using equation (16)	
12	Update the channel population **D**_**k**_	
13	If (number of cycles > required NoC) go to step 14 Else go to step 9	

*Level 3: in this level, the discrete FL-OLMPSO algorithm is used for estimating the data symbols*		
14	The global best particle of the data population is chosen and update the velocity: W_him(n)_ = **FLC** (LI, GI, W_him(n−1)_)	
15	Update position of each particle of data population Compute the mutant operator (MO) M_od_(i) = ∑_*j*=1_^*k*^*W*_dij_/*k* D_pim_(n) = D_pim_(n−1) + M_od_(i) ∗ rand()	
16	Compute the fitness of particles of data population using (16)	
17	Update the data population **D**_**p**_	
18	If (number of cycles > required NoC) go to step 20 Else go to step 14	

*Level 4: next sample of the received signal is taken and execution goes to level 2*		
19	Stop	

**Table 2:** I/O variables membership functions used in the proposed FL-OLMPSO.

S. no.	Input variables	Mathematical representation of membership functions (MFs)	Graphical representation of MFs
1	LocalInt ((*μ*_localint_(*l*))	$\mu_{\text{localint},\text{small}}\left(l\right)=\left\{\begin{array}{cc} \left(0.35-l\right)/0.35, & 0\leq l\leq 0.35\\ 0, & \text{else} \end{array}\right\}$ $\mu_{\text{localint},\text{medium}}\left(l\right)=\left\{\begin{array}{cc} \left(l/0.35\right), & 0\leq l\leq 0.35\\ \left(0.65-l\right)/0.3, & 0.35\leq l\leq 0.65 \end{array}\right\}$ $\mu_{\text{localint},\text{large}}\left(l\right)=\left\{\begin{array}{cc} \left(l-0.35\right)/0.35, & 0.35\leq l\leq 0.65\\ \left(1-l\right)/0.35, & 0.65\leq l\leq 1 \end{array}\right\}$ $\mu_{\text{localint},\text{v}.\text{large}}\left(l\right)=\left\{\begin{array}{cc} \left(l-0.65\right)/0.35, & 0.65\leq l\leq 1\\ 0, & \text{else} \end{array}\right\}$	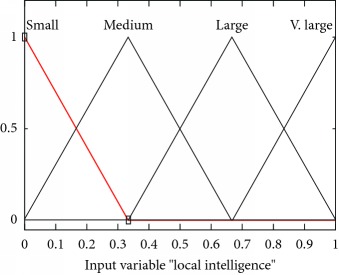

2	GlobalInt (*μ*_globalInt_(*G*))	$\mu_{\text{globalint},\text{small}}\left(G\right)=\left\{\begin{array}{cc} \left(0.35-g\right)/0.35, & 0\leq g\leq 0.35\\ 0, & \text{else} \end{array}\right\}$ $\mu_{\text{globalint},\text{medium}}\left(G\right)=\left\{\begin{array}{cc} g/0.35, & 0\leq g\leq 0.35\\ \left(0.65-g\right)/0.3, & 0.35\leq g\leq 0.65 \end{array}\right\}$ $\mu_{\text{globalint},\text{large}}\left(G\right)=\left\{\begin{array}{cc} \left(0.35-g\right)/0.35, & 0\leq g\leq 0.35\\ 0, & \text{else} \end{array}\right\}$ $\mu_{\text{globalint},\text{v}.\text{large}}\left(G\right)=\left\{\begin{array}{cc} g/0.35, & 0\leq g\leq 0.35\\ \left(0.65-g\right)/0.3, & 0.35\leq g\leq 0.65 \end{array}\right\}$	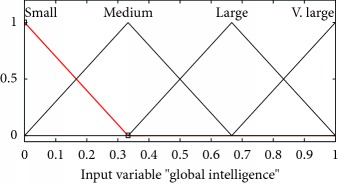

3	Prevelocity (*μ*_prevelocity_(*PV*))	$\mu_{\text{prevelocity},\text{v}.\text{slow}}\left(PV\right)=\left\{\begin{array}{cc} \left(0.5-pv\right)/0.5, & 0\leq pv\leq 0.5\\ 0, & \text{else} \end{array}\right\}$ $\mu_{\text{prevelocity},\text{slow}}\left(PV\right)=\left\{\begin{array}{cc} pv/0.25, & 0\leq pv\leq 0.25\\ \left(1-pv\right)/0.75, & 0.25\leq pv\leq 1 \end{array}\right\}$ $\mu_{\text{prevelocity},\text{medium}}\left(PV\right)=\left\{\begin{array}{cc} \left(pv-1\right)/0.75, & 0.25\leq pv\leq 1\\ \left(1.5-pv\right)/0.5, & 1\leq pv\leq 1.5 \end{array}\right\}$ $\mu_{\text{prevelocity},\text{fast}}\left(PV\right)=\left\{\begin{array}{cc} \left(pv-1.5\right)/0.5, & 1\leq pv\leq 1.5\\ \left(2-pv\right)/0.5, & 1.5\leq pv\leq 2 \end{array}\right\}$ $\mu_{\text{prevelocity},\text{v}.\text{fast}}\left(PV\right)=\left\{\begin{array}{cc} \left(pv-2\right)/0.5, & 1.5\leq pv\leq 2\\ 0. & \text{else} \end{array}\right\}$	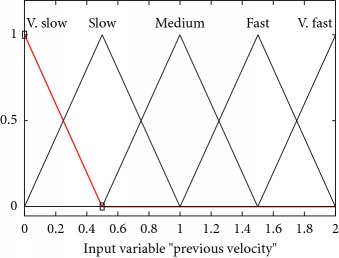

4	Output, UV (*μ*_output,*UV*_(*UV*))	$\mu_{UV,\text{v}.\text{slow}}\left(UV\right)=\left\{\begin{array}{cc} uv/0.2, & 0\leq uv\leq 0.1\\ \left(0.4-uv\right)/0.2, & 0.1\leq uv\leq 0.2 \end{array}\right\}$ $\mu_{UV,\text{slow}}\left(UV\right)=\left\{\begin{array}{cc} \left(uv-0.3\right)/0.2, & 0.1\leq uv\leq 0.25\\ \left(0.5-uv\right)/0.2, & 0.25\leq uv\leq 0.4 \end{array}\right\}$ $\mu_{UV,\text{medium}}\left(UV\right)=\left\{\begin{array}{cc} \left(uv-0.8\right)/0.2, & 0.3\leq uv\leq 0.45\\ \left(1-uv\right)/0.2, & 0.45\leq uv\leq 0.6 \end{array}\right\}$ $\mu_{UV,\text{fast}}\left(UV\right)=\left\{\begin{array}{cc} \left(uv-0.8\right)/0.2, & 0.5\leq uv\leq 0.65\\ \left(1-uv\right)/0.2, & 0.65\leq uv\leq 0.8 \end{array}\right\}$ $\mu_{UV,\text{v}.\text{fast}}\left(UV\right)=\left\{\begin{array}{cc} \left(uv-0.8\right)/0.2, & 0.7\leq uv\leq 0.85\\ \left(1-uv\right)/0.2, & 0.85\leq uv\leq 1 \end{array}\right\}$	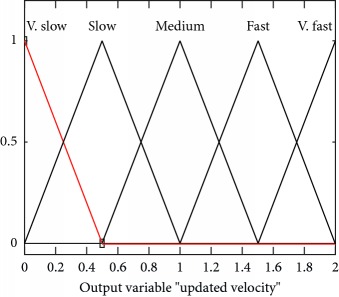

**Table 3:** Lookup table for the proposed FL-OLMPSO.

Rules	Local intelligence	Global intelligence	Previous velocity	Updated velocity
1	Small	Small	Very slow	Very slow
2	Small	Small	Slow	Slow
3	Small	Small	Medium	Slow
4	Medium	Small	Slow	Slow
5	Medium	Medium	Medium	Medium
6	Medium	Small	Medium	Medium
7	Medium	Medium	Very fast	Fast
8	Large	Large	Medium	Fast
9	Very large	Large	Very fast	Very fast
10	Very large	Very large	Very fast	Very fast

## Data Availability

The simulation data used to support the findings of this study are available from the corresponding author upon request.
